# Modifications in Upper Airway Collapsibility during Sleep Endoscopy with a Mandibular Positioner: Study in Snorers and Obstructive Sleep Apnea Patients

**DOI:** 10.3390/jcm13051184

**Published:** 2024-02-20

**Authors:** Patricia Fernández-Sanjuán, Marta Alcaraz, Gabriela Bosco, Nuria Pérez-Martín, Marta Morato, Rodolfo Lugo, Juan José Arrieta, Jaime Sanabria, Marcos Ríos-Lago, Guillermo Plaza

**Affiliations:** 1 Sleep Respiratory Disorders Unit, Hospital Universitario La Zarzuela, 28023 Madrid, Spain; patriciafsanjuan@yahoo.es (P.F.-S.); maria.bosco@salud.madrid.org (G.B.); n.perezmartin@hotmail.com (N.P.-M.); 2 Universidad Rey Juan Carlos, 28002 Madrid, Spain; 3 Department of Otolaryngology, Hospital Universitario La Moraleja, 28050 Madrid, Spain; malfue@hotmail.com; 4 Department of Otolaryngology, Hospital Universitario de Fuenlabrada, 28942 Madrid, Spain; 5 Department of Otolaryngology, Hospital Universitario La Zarzuela, 28023 Madrid, Spain; 6 Department of Otolaryngology, Hospital Quirónsalud San José, 28002 Madrid, Spain; marta37003@gmail.com; 7 Department of Otolaryngology Head and Neck Surgery, Hospital San José, Monterrey 64718, Mexico; rodo_lugo@me.com; 8 Department of Stomatology, Hospital Universitario Fundación Jiménez Díaz, 28040 Madrid, Spain; jjarrietab@yahoo.es; 9 Department of Otolaryngology Head and Neck Surgery, Hospital Universitario Fundación Jiménez Díaz, 28040 Madrid, Spain; jsanabria@fjd.es; 10 Department of Basic Psychology II, Faculty of Psychology, UNED—Universidad Nacional de Educación a Distancia, 28040 Madrid, Spain; mrios@psi.uned.es

**Keywords:** obstructive sleep apnea (OSA), collapsibility, mandibular advancement device (MAD), titratable positioner, Drug-Induced Sleep Endoscopy (DISE)

## Abstract

Background: Mandibular advancement devices (MADs) are an effective treatment for patients with sleep-related breathing disorders, with variable response. Increasingly more research points to the predictive value of Drug-Induced Sleep Endoscopy (DISE) in patient selection. This study aims to analyze the changes in upper airway collapsibility using a titratable MAD simulator during DISE. Methods: This study included 104 patients with simple snoring and obstructive sleep apnea (OSA). The VOTE scale was used to assess the presence of collapses during the DISE both without and with the MAD simulator. Results: In snorers, there was a decrease in collapses at the level of the soft palate and oropharynx when the advancement was achieved. Patients with mild OSA also showed a decrease in collapses at the base of the tongue. Patients with moderate/severe OSA exhibited significant amelioration at all levels. The levels at which there were residual collapses despite the maneuver were, in order, the velopharynx, oropharynx, epiglottis, and tongue. Conclusions: The MAD simulator reduces collapsibility at all levels and in all severity groups. Residual collapses suitable for combined treatments were able to be identified. This highlights the need for individualized patient selection, as upper airway collapsibility exhibits variable improvement or worsening with the MAD simulator regardless of the severity of the condition.

## 1. Introduction

Sleep-related breathing disorders, snoring, and sleep apnea represent a growing concern in public health due to their prevalence and significant impact on individual quality of life. Snoring prevalence varies according to various studies, reaching 86% depending on the method of measurement used and the population analyzed, showing a higher prevalence in the male compared to the female gender [1,2]. It is commonly characterized as a harsh and vibratory sound during sleep, caused by partial obstruction principally during inspiration. Despite snoring often being trivialized, it may be the initial sign of a spectrum of sleep-disordered breathing, which spans from partial airway collapse and mild upper airway resistance to total airway collapse and severe obstructive sleep apnea (OSA). There is increasing evidence that snoring is linked to several health issues. In fact, it has been independently associated with increased odds of metabolic syndrome, especially in women [3]. In addition, snoring is associated with a modest but significantly increased risk of cardiovascular disease (independent of age), smoking, Body Mass Index (BMI), and other cardiovascular risk factors [4].

Similarly, substantial methodological heterogeneity exists in studies that investigate the population prevalence of OSA, and there is a resultant wide variation in the reported prevalence. The Senaratna et al. [5] study concluded that the overall prevalence of any form of OSA ranged from 9% to 38% in the general adult population, from 13% to 33% in men, and from 6% to 19% in women. On average, the gender ratio for middle-aged individuals is 2-3/1 for men and women, respectively. Furthermore, this prevalence increases with age, tripling in the elderly population compared to middle-aged individuals [2,6], and increases with obesity which is considered a major risk factor for the development and progression of OSA [7]. Patients with OSA experience apneas and/or hypopneas during sleep, leading to intermittent hypoxemia and changes in intrathoracic pressure that can trigger cardiovascular and metabolic consequences. Additionally, the disruption of sleep architecture can result in neuropsychological and psychiatric issues. All these factors lead to a decreased quality of life, which is associated with excess mortality, making it a significant public health concern [8].

Currently, the treatment of OSA is multidisciplinary, including common lifestyle measures such as sleep hygiene, exercise, and weight loss [8]. Although continuous positive airway pressure (CPAP) therapy is the most common treatment, patients may benefit from other therapies, such as various surgical treatments, myofunctional therapy, positional therapy, and/or the use of mandibular advancement devices (MADs) [8,9].

The first publications on the use of MADs in OSA appeared around 1980 [10,11]. There is now evidence that MADs are an effective treatment for many OSA patients [11,12,13,14] and are becoming an increasingly popular alternative, primarily directed at patients with snoring, mild to moderate OSA, and those with severe OSA who reject or cannot tolerate CPAP [13,15]. MADs aim to stabilize and advance the mandible and hyoid bone to a forward position, preventing posterior jaw rotation when sleeping in the supine position and upper airway (UA) obstruction. Thus, this jaw advancement aims to increase the upper airway space predominantly at the velopharynx level, increase tension in the lateral pharyngeal walls, and reduce their collapsibility. It also advances the tongue, separating it from the posterior pharyngeal wall, and contributes to the tensioning of its musculature, reducing tissue vibration and consequently decreasing snoring [13,15]. MADs can be used in isolation, in combination, or alternated with other treatment modalities that, when used alone, do not achieve the desired therapeutic goal [16,17], or to reduce the pressure exerted during CPAP therapy, improving its adherence [18,19].

Various predictors of success for selecting patients who could benefit from MAD treatment have been described in the literature. Among anthropometric parameters, noteworthy factors include being young, female, having a low BMI, or having a smaller neck circumference. Among polysomnographic parameters, potential responders are those with a lower Apnea–Hypopnea Index (AHI), supine-dependent OSA, or a high minimum oxygen saturation [20]. On the other hand, predictors of failure include the need for high positive airway pressure [21], a narrower airway, or a long soft palate [22]. With the same objective of analyzing modifications in the UA, studies have been published that analyze changes using three-dimensional volumetric studies with imaging tests such as computed tomography (CT) or cone beam computed tomography (CBCT) in awake patients using a MAD in situ with controversial results. Some of these studies [23,24,25,26] concluded that there is an increase in UA volume when the patient uses a MAD in situ, which correlates with the improvement in the AHI in PSG. Conversely, Shi et al. [27] observed that these increases in UA dimensions in CBCT with MAD use were not significantly different between responders and non-responders. All these studies have the limitation of being conducted in awake patients using a MAD in situ to observe changes in the UA in imaging tests.

On the other hand, it has been shown that natural sleep does not significantly differ from drug-induced sleep regarding respiratory parameters [28] and upper airway obstruction sites [29]. Therefore, conducting a Drug-Induced Sleep Endoscopy (DISE) to evaluate the upper airway in OSA patients appears appropriate, as it provides the most information about airway collapsibility [30]. The study of the behavior of the airway under sedation allows for the development of more precise and personalized approaches for the diagnosis and treatment of respiratory disorders. It helps anticipate the likelihood of a positive or negative response to certain treatments, enabling more informed management. DISE shows a relevant influence on the location of the recommended treatment. Thus, a change in success rates of non-CPAP therapy in OSA and snoring individuals appears possible [31]. Concerning MADs, different mandibular advancement maneuvers have been described during a DISE to attempt to identify patients who respond to this therapy [32]. The objective of these maneuvers is to assess whether simulating MAD treatment during sedation is expected to stabilize one or multiple levels of the upper airway in patients with obstructive events or snoring during their physiological sleep. During this assessment, the range of anteroposterior mandibular movement should be considered to avoid exceeding it during the DISE, beyond tolerable limits, and thus avoid generating false expectations about the tolerance and effectiveness of MAD treatment in the studied patient. Furthermore, all levels of the upper airway should be explored, and the response to mandibular advancement at each level should be evaluated [33]. In this regard, optimizing and standardizing the mandibular advancement maneuver during a DISE to eliminate inter-examiner bias is desirable. Since its inception, manual mandibular traction maneuvers, simulation bites, or provisional devices that mimic the effect of MADs during a DISE have been proposed to predict the individual response in the patient [32] (Table 1).

According to some publications [36,41,50], performing manual maneuvers is not considered an appropriate technique for selecting patients eligible for MAD therapy, as it may lead to awakenings during the DISE and may not accurately replicate the real effect of a MAD.

The aim of the present study is to objectively evaluate modifications in the collapsibility of the upper airway during a DISE with a stable, titratable, and reproducible maneuver that mimics the effect of a MAD [49].

## 2. Materials and Methods

### 2.1. Participants

A prospective observational descriptive study was designed, including patients diagnosed with snoring and OSA from the Otorhinolaryngology and Dental Sleep Medicine departments of the University Hospitals of Fuenlabrada, Fundación Jiménez Díaz, and La Zarzuela in Madrid, Spain.

The Medical Ethics Committee of the Hospital Universitario de Fuenlabrada approved the study protocol in accordance with the Declaration of Helsinki (APR 20/30), and signed informed consent was obtained from all participants. Data on the study subjects were collected and stored anonymously to protect personal information.

The sample comprised 104 patients who met the following inclusion and exclusion criteria. The inclusion criteria were the following: age over 18 years; a diagnosis of simple snoring or OSA; complete otorhinolaryngological history and examination, including an upper airway endoscopy while awake during an office visit; complete dental history; and signed consent. Exclusion criteria were prior surgery for OSA, propofol allergy, an insufficient number of teeth or poor periodontal health, severe temporomandibular joint pathology, mandibular anteroposterior movement < 5 mm, craniofacial abnormalities, or treatment with muscle relaxants. The sample size was calculated using the G*Power program (Version 3.1.9.6) [51]. Based on the effect sizes available in the literature and a pilot study, a group of 100 participants was estimated to achieve a contrast power of 80% with a 95% confidence level.

### 2.2. Procedure

Patient history was collected, including sociodemographic data: age, sex, and biometric data such as weight, height, and BMI. All patients were evaluated using standard overnight polysomnography, interpreted according to the criteria established by the American Academy of Sleep Medicine (2011) and a level 2 respiratory polygraphy with cardiac and respiratory variables analysis. In all cases, the AHI was determined while considering the number of apneas and hypopneas per hour.

**Figure 1:**
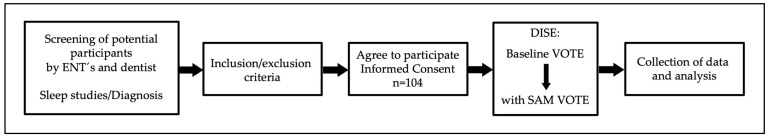
Study flowchart.

All subjects underwent a DISE according to the protocol established in the European position paper [30] with the participation of an anesthesiologist, an otorhinolaryngologist, and a dentist. Standard monitoring was performed, and continuous propofol infusion at 2% with a target concentration of 2 ng/mL was used, with progressive increases of 0.2 to 0.5 ng/mL when required to maintain the desired sedation level. The sedation level was monitored using the Bispectral Index (BIS) (BIS Quatro^®^, Covidien ILc, Mansfield, MA, USA) to maintain the BIS levels between 50 and 70. Vasoconstrictors and topical anesthetics were not used. The video-flexible endoscope (MACHIDA ENT-30PIII, Chiba, Japan) was introduced and positioned in the nasopharynx, where the otorhinolaryngologist waited for the appearance and visualization of two cycles of snoring, apnea, and breathing. Then, the upper airway was examined up to the larynx. The VOTE scale, as described by Kezirian et al. [52], was used to assess the degree of collapses at different levels. This classification defines the collapses with a number: 0, no obstruction (no vibration); 1, partial obstruction (vibration); and 2, complete obstruction (collapse). Regarding the levels of collapse, four levels are studied: the velopharynx (V), oropharynx (O), base of the tongue (T), and epiglottis (E). Measurements were taken twice during the DISE: first, during the baseline condition without any device for simulation of MAD treatment, then when the device for advancing the mandible and simulating a MAD treatment was introduced in the mouth. The progressive jaw advancement was produced following the DISE-SAM protocol [49]. A second measure using the VOTE scale was taken (with-SAM condition) (see Figure 1).

**Figure 2:**
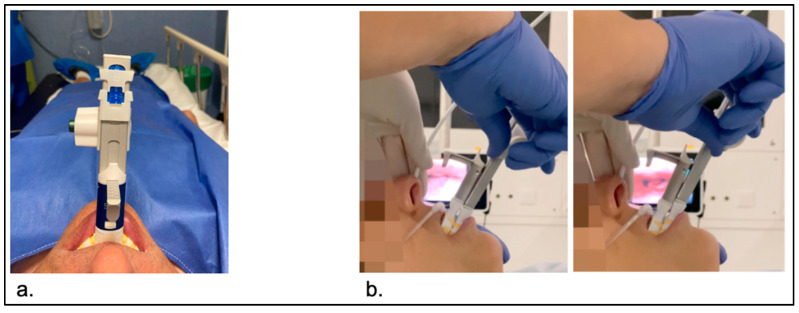
(**a**) SAM device at maximum comfortable protrusion. (**b**) Jaw without and with mandibular traction, and visualization of the improvement in collapses on the screen.

For simulating MAD treatment, a SAM device was used that was specifically designed for this purpose [49]. It is a titratable tool that allows the operator perform controlled maneuvers with high precision (in the mm range) during a DISE, starting from a neutral/retrusive position to reach the optimal position where, if the patient responds, apnea/snoring are eliminated (Video S1). The device consists of two parts: a reusable body and single-use trays to be inserted into the patient’s mouth. The trays can be inserted or removed independently of the device’s body, facilitating mouth insertion or removal at any time. Before sedating the patient, the SAM device was personalized to the patient’s dental arches using polyvinylsiloxane dental impression material (Video S2). The patient was asked to move the jaw freely forward and backward before sleep induction. The maximum retrusive movement and maximum protrusive positions were delimited by the operator with markers on a scale to avoid exceeding them during sedation (Video S3). Once the upper airway was studied in the baseline condition, the SAM device with customized trays for dental arches was inserted, and the operator changed the mandible’s position by manipulating a manually operated knob. The position can be changed as many times as necessary without removing the device from the patient’s mouth, allowing for the mandibular position to be fixed in the position of interest (Figure 2). The examination was conducted in the supine position rather than in lateral decubitus positions to avoid the potential influence that changes in body’s position might have on airway modifications.

All recorded data were included in a database for subsequent analysis.

### 2.3. Statistical Analysis

Statistical analyses were performed in three main steps.

First, the presence of collapses and their degree (complete: 2; partial: 1; and no collapse: 0) at different levels of the upper airway were analyzed both at baseline (without the SAM device) and under with-SAM conditions using McNemar’s Z statistic. This analysis was performed for the whole group of participants.

Then, the participants were divided into four severity groups. A repeated measures Wilcoxon Z analysis was used with the aim of detecting differences between the VOTE measures in the baseline condition and the results produced by mandibular advancement (with-SAM condition) at these four levels of the UA in each of the four severity groups.

Finally, Venn diagrams were employed to study the presence of complete collapses at the VOTE levels at baseline and under with-SAM conditions. This analysis was performed separately: first for the whole group and then for the four severity groups.

A significance level of *p* < 0.05 was set to consider existing differences as significant. The SPSS statistical software package, version 27, was used for all analyses.

## 3. Results

The sample consisted of 104 patients, with a mean age of 48.75 ± 11.5 years, and 80.8% (n = 84) were male. The mean BMI was 27.63 ± 3.75 kg/m^2^.

**Figure 3:**
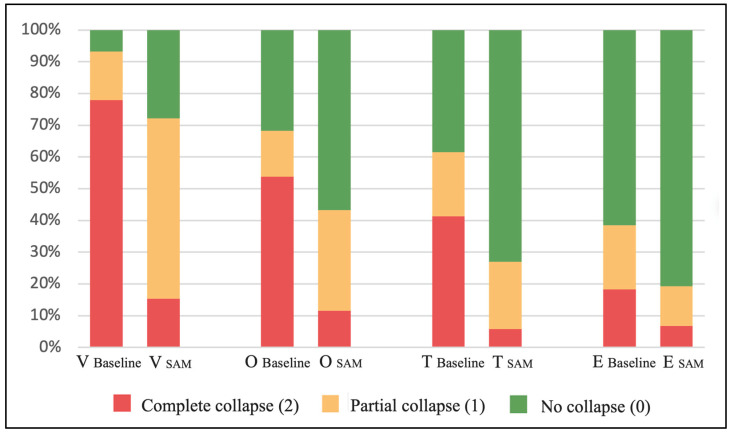
Percentage of patients with complete collapse, partial collapse, or no collapse at each VOTE level in baseline condition and with mandibular advancement simulation using SAM.

As shown in Table 2 and Figure 3, a change in the patient distribution between the baseline condition and the with-SAM condition was observed for each of the collapse degrees and VOTE levels. The percentage of patients with complete collapses decreased at all upper airway levels (*p* < 0.001 in all levels), while the percentage of patients without collapses increased in all four levels as well (*p* < 0.001 in all levels). Regarding partial collapses, changes were only detected at the V and O levels (*p* < 0.001 and *p* = 0.004, respectively), with no significant changes in the percentage of patients who modified their collapsibility in T (Z = −0.18; *p* = 0.86) and E (Z = 1.71; *p* = 0.09).

When participants were subsequently divided into four severity subgroups based on their AHI scores, the following characteristics were obtained for each group: simple snoring with AHI < 4.9 (n = 17; 16.3%), mild OSA with AHI 5–14.9 (n = 22; 21.2%), moderate OSA with AHI 15–29.9 (n = 23; 22.1%), and severe OSA with AHI > 30 (n = 42; 40.4%).

**Figure 4:**
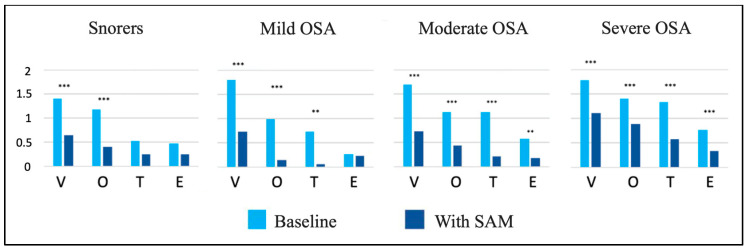
Changes in the VOTE levels at baseline and with SAM device within the four severity groups. V: velopharynx; O: oropharynx; T: base of the tongue; E: epiglottis; **: *p* < 0.01; ***: *p* < 0.001; ns: not significant.

Differences were detected between the VOTE measures in the baseline condition and the results produced by the mandibular advancement (with-SAM condition) at these four levels. The results obtained are presented in Table 3 and Figure 4 for the four severity groups.

**Figure 5:**
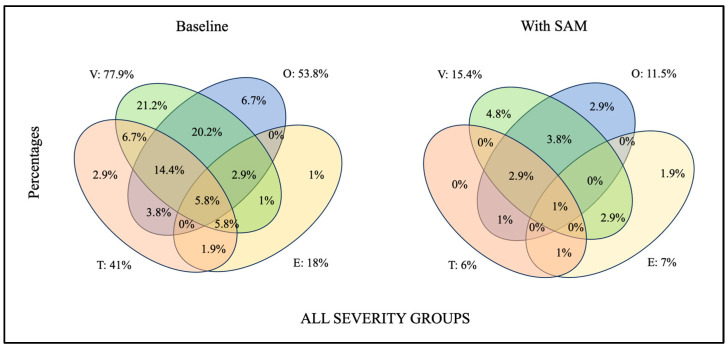
Venn diagram illustrating the presence in percentages of complete collapses at different VOTE levels and the existence of multilevel complete collapses at baseline and with SAM device in the whole sample (V: velopharynx; O: oropharynx; T: base of the tongue; E: epiglottis).

**Figure 6:**
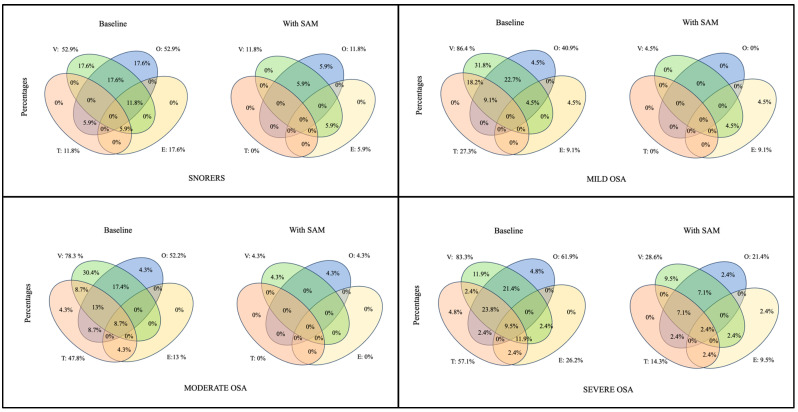
Venn diagram illustrating the presence in percentages of complete collapses at different VOTE levels and the existence of multilevel complete collapses at baseline and with SAM device for every severity group independently. (V: velopharynx; O: oropharynx; T: base of the tongue; E: epiglottis).

Finally, the presence of complete collapses was studied at all VOTE levels and in all possible combinations. This analysis was performed under both baseline and with-SAM conditions for the whole group and independently for each severity group. The results are displayed in Figure 5 (all severity groups) and Figure 6 for each severity group.

## 4. Discussion

MADs have become an alternative treatment for patients with snoring and/or OSA, and their use is increasing due to the good results shown in many patients [8,13,19]; however, as with other treatments, the adaptation of a MAD is not effective in all cases since the origin of OSA is multifactorial [22]. Moreover, the use of a MAD can lead to the worsening of the baseline condition. It is known that the greater the severity, the lower the expected response, but on the other hand, a complete response can be achieved regardless of the severity for many patients [13].

In the present study, modifications in the collapsibility of the UA were analyzed in patients with sleep-disordered breathing during a DISE with a titratable MAD simulator, the *Selector de Avance Mandibular* (SAM). The objective evaluation of the mandibular advancement simulation is expected to contribute to the selection of patients suitable for MAD treatment or for the selection of a combination of therapies. To the best of our knowledge, this is the first study to perform an analysis of the changes observed at each VOTE level, based on the degree of severity of the sleep-disordered breathing, and using a titratable device. Also, for the first time, UA collapsibility in simple snoring has been studied during a DISE.

Differing from investigations using imaging tests such as CBCT and CT scans that focused on examining patients’ UA during wakefulness [23,24,25,26,53], in the current study, modifications were monitored during a DISE which is a validated technique representative of physiological sleep [29,34,54,55,56,57]. Our results are in line with other studies that consider DISE to be a good technique for evaluating the location and pattern of UA collapses, as well as the capability of mandibular advancement to identify candidates for MAD treatment [36,43,44,50,56,58].

The initial publications mentioning the utility of DISE for MAD patient selection described manual maneuvers to mimic the effect of a MAD [34]. Today, it is known that manual maneuvers are discouraged because it may fall short and fail to identify a potential responder patient. Also, a manual maneuver can produce an exaggerated response in the hypopharynx that does not correlate with the effect of a MAD; it could induce arousals, and in addition, the increase in the vertical dimension produced by the MAD is not considered [30,36,43,49]. To address these issues and standardize the maneuver, the SAM device allows for the simulation of the effect of a MAD with a reproducible and quantifiable maneuver that mimics the size of a MAD-eliminating inter-examiner bias.

When analyzed as a group, the present results show that when implementing the maneuver with the SAM device, there are improvements observed across all levels of the VOTE scale. Complete collapses decrease, either transforming into partial collapses or completely disappearing, and there is an increase in the number of levels with no collapses. With this personalized information, targeted combined therapies could be suggested to alleviate residual collapses when complete stabilization is not expected. Vroegop et al. [36] found that in the baseline situation, collapses at the palate level were the most frequent, followed by at the base of the tongue and epiglottis, while oropharyngeal collapses were the least frequent. The results of the present study coincide with the observation that as a group, the level at which collapses most frequently occur in the baseline situation is the velum; however, it was observed that the next most common level for collapses was the oropharynx, followed by the base of the tongue and epiglottis.

When the modifications in the different severity groups were analyzed, the present study revealed that as the severity of the respiratory disorder increases, collapsibility also increases in the baseline situation at all levels. In snorers, complete collapses were mainly found in the soft palate and oropharynx. In mild OSA patients, collapses were also present at the base of the tongue, and in moderate/severe OSA patients, collapses were present at all levels, including the epiglottis. With the SAM device, an improvement in snorers occurs in the V and O, so if there is residual snoring despite the use of a MAD, the therapy should be aimed at alleviating collapses of the velo- or oropharynx. As the severity increases, this improvement is also observed in the T and E. In other words, the improvement offered by a MAD could occur at all levels and across different severity groups. In moderate/severe OSA patients, residual collapses can be present at any level, hence the importance of an examination under induced sleep to better direct patients’ therapy in more severe groups as it allows for a personalized approach.

As in other studies, an analysis of the presence and modifications in multilevel collapses was conducted. All isolated and multilevel collapses improved with the use of the SAM device except for some related to the E. In the supine position, secondary epiglottal collapses at the base of the tongue improved when the tongue improved with the use of a MAD in all severity groups. When the collapse was primary, it only improved in snorers in 50% of the patients who used the SAM device; however, it did not improve in mild and severe cases. Additionally, there is a small group of patients (2.4%) in whom mandibular advancement can cause deterioration at this level. This suggests that mandibular advancement not only does not improve some collapses of the E but can also cause the epiglottis to collapse when it does not in the baseline situation. This finding can explain the deterioration for some patients when using a MAD in the supine position. For these patients, evidence shows that this epiglottal collapse could be treated with positional therapy [59]. When studying multilevel collapses, Vroegop et al. [36] noted frequent combinations of collapse levels, with the most common collapse pattern being a combination of palatal and base-of-tongue collapses (34.4%). In our study, multilevel collapses were also studied, with the velum and oropharynx being the most common (20.2%), followed by the velum, oropharynx, and base-of-tongue collapse (14.4%). It should be noted that our sample included snoring patients (16.3% of the patients), which could influence the results as they showed more collapsibility at the velo- and oropharynx at baseline. Using the SAM device, a considerable decrease in the number of patients with multilevel collapses (combinations of two, three, and four collapses) was observed. Only 5.8% exhibited complete collapses at all levels, which reduced to 1% of patients in whom no complete collapse was resolved using the SAM device. Cavaliere et al. [44], using a comfortable maximum protrusion bite, found combined collapses in 65% of the patients. In the present study, the presence of combined collapses in the baseline situation was 60.6%, slightly lower possibly due to the inclusion of snorers in our study. With the use of the SAM device in situ, the combined collapses decreased to 11.6%. This suggests that if a MAD cannot be used as a sole treatment, it could still be an important tool for combined therapy by contributing to the relief of collapsibility at all levels. In addition, for the first time, a combination of collapses was analyzed by severity group independently, with the most frequent combination being the V-O in snorers and mild and moderate OSA patients, and a combination of V-O-T in severe OSA patients.

In the present study, as a group, the levels responsible for residual complete collapses that could be responsible for a poor response are, in order, V, O, E, and T, and, to a lesser extent, persistent multilevel collapses; however, when analyzed by severity groups, the most frequent residual complete collapses that occurred in snorers were in the V and O, and in the combination of the V-O and V-E. In mild OSA patients, this was observed in E and the combination of V-E, while in moderate cases, it occurred in the V and O. For severe cases, the highest magnitude of residual complete collapses was seen in the V, O, T, and E, as well as in the combination of V-O and V-O-T (see Figure 5). Similarly, Vonk et al. [43] observed, using a provisional MAD, that the group of patients identified as unsuitable candidates was due to residual complete retropalatal collapses, residual complete hypopharyngeal collapses, and, to a lesser extent, persistent multilevel collapses.

One of the strengths of this study is the ability to standardize the mandibular advancement simulations, with the option to customize it by using a titratable device. Until now, provisional devices with a single position to explore have been employed. This feature can help identify potential deteriorations in collapsibility, such as what may occur at the epiglottis for some patients when inducing larger advancements. Among the study limitations, it should be noted that the examinations were performed at different centers following European consensus recommendations on DISEs to standardize criteria. The same dentist was always present, performing the mandibular advancement maneuvers, but different otolaryngologists and anesthesiologists with extensive experience in the technique participated. Secondly, the assessment of baseline collapsibility as well as the modifications resulting from the incorporation of the SAM device is subjective, and there may be some inter-examiner variability. Lastly, it should be noted that these sedations were carried out with propofol, so REM sleep cannot be visualized.

## 5. Conclusions

The MAD simulator allows for the objective assessment of modifications in the upper airway in sedated patients. A reduction in collapsibility at all levels of the UA is observed and in all severity groups, leading to a general decrease in the number of complete collapses. This procedure could detect residual collapses, making it possible to identify patients suitable for combined treatments. To summarize, in snoring and mild OSA patients, a combination of therapies could be employed to alleviate collapses of the soft palate and oropharynx, and in more severe OSA patients, a combination of therapies aimed at any level should be considered, hence the importance of a personalized study under sedation. The levels primarily associated with residual collapses despite the use of the MAD simulator are the velopharynx and oropharynx, followed by the epiglottis and tongue. It is worth noting that in some cases, the use of a MAD could cause primary collapses of the epiglottis in the supine position, which could result in deterioration compared to the baseline. This highlights the need for individualized patient selection, as upper airway collapsibility exhibits variable improvement or worsening with MAD simulation regardless of the severity.

## Figures and Tables

**Table 1:** Publications with mandibular advancement simulation using different jaw advancement maneuvers during Drug-Induced Sleep Endoscopy (modified from reference [32]).

AUTHOR	YEAR	n	MANEUVER
**Johal et al.** [34]	2005	19	Manual advancement maneuver and mad in situ
**Johal et al.** [35]	2007	120	4–5 mm advancement maneuver performed by the anesthesiologist
**Vroegop et al. and Vanderveken et al.** [36]	2013	200	Simulation bite
**De Corso et al.** [37]	2015	65	Less than 5 mm manual advancement maneuver
**Huntley et al.** [38]	2018	20	Manual advancement maneuver
**Beelen et al.** [39]	2018	200	Manual advancement maneuver (jaw thrust)
**Kastoer et al.** [40]	2018	10	Remotely controlled mandibular positioner
**Park et al.** [41]	2019	40	Modified mandibular advancemen maneuver. 50–75% manual advancement
**Carrasco et al.** [42]	2019	-	Modified Esmarch maneuver
**Vonk et al.** [43]	2020	63	Manual advancement maneuver and boil and bite mad
**Cavaliere et al.** [44]	2020	66	Simulation bite
**Koutsourelakis et al.** [45]	2021	49	Manual advancement maneuver
**Veugen et al.** [46]	2021	70	Manual advancement maneuver
**Kazemeini et al.** [47]	2022	10	Remotely controlled mandibular positioner
**Bosschieter et al.** [48]	2022	94	Manual advancement maneuver and boil and bite mad
**Fernández-Sanjuán et al.** [49]	2022	161	Mandibular titratable positioner (SAM)
**Van den Bossche et al.** [50]	2023	56	Manual advancement maneuver and mad in situ

**Table 2:** Changes in the proportion of patients between the baseline condition and the with-SAM condition for each of the collapse grades and VOTE levels.

	V Baseline	V SAM	Z	*p*	O Baseline	O SAM	Z	*p*	T Baseline	T SAM	Z	*p*	E Baseline	E SAM	Z	*p*
**Complete** **collapse (2)**	77.9	15.4	8.06	***	53.8	11.5	6.63	***	41.3	5.8	6.08	***	18.3	6.7	3.21	***
**Partial** **collapse (1)**	15.4	56.7	−5.6	***	14.4	31.7	−2.92	**	20.2	21.2	−0.18	ns	20.2	12.5	1.71	ns
**No** **collapse (0)**	6.7	27.9	−4.69	***	31.7	56.7	−5.1	***	38.5	73.1	−6	***	61.5	80.8	−4.26	***
	100	100			100	100			100	100			100	100		

V: velopharynx; O: oropharynx; T: base of the tongue; E: epiglottis; Z: McNemar’s Z statistic; **: *p* < 0.01; ***: *p* < 0.001; ns: not significant.

**Table 3:** Changes in the VOTE scale within the four severity groups.

Severity		Baseline	With SAM	Z Wilconxon	*p*
**Snorers**	V	1.41	0.65	−3.13	***
	O	1.18	0.41	−2.6	***
	T	0.53	0.24	−1.63	ns
	E	0.47	0.24	−1.63	ns
**Mild OSA**	V	1.81	0.73	−4.02	***
	O	1	0.14	−3.27	***
	T	0.73	0.05	−2.87	**
	E	0.27	0.23	−0.27	ns
**Moderate OSA**	V	1.7	0.74	−3.95	***
	O	1.13	0.43	−3.36	***
	T	1.13	0.22	−3.52	***
	E	0.17	0.17	−3	**
**Severe OSA**	V	1.79	1.12	−4.77	***
	O	1.4	0.88	−4.3	***
	T	1.33	0.57	−4.46	***
	E	0.76	0.33	−3.45	***

V: velopharynx; O: oropharynx; T: base of the tongue; E: epiglottis; **: *p* < 0.01; ***: *p* < 0.001; ns: not significant.

## Data Availability

Data are contained within the article.

## Supplementary Materials

The following supporting information can be downloaded at: https://www.mdpi.com/article/10.3390/jcm13051184/s1, Video S1: Titratable tool. Video S2: Personalized jaw opening. Video S3: Maximum retrusive movement and maximum protrusive positions.
